# The Role of Transjugular Intrahepatic Portosystemic Shunt for the Management of Ascites in Patients with Decompensated Cirrhosis

**DOI:** 10.3390/jcm13051349

**Published:** 2024-02-27

**Authors:** Giulia Iannone, Enrico Pompili, Clara De Venuto, Dario Pratelli, Greta Tedesco, Maurizio Baldassarre, Paolo Caraceni, Giacomo Zaccherini

**Affiliations:** 1 Department of Medical and Surgical Sciences, University of Bologna, 40138 Bologna, Italy; giuliaiannone21@gmail.com (G.I.); enrico.pompili3@unibo.it (E.P.); clara.devenuto@studio.unibo.it (C.D.V.); dario.pratelli@studio.unibo.it (D.P.); greta.tedesco2@unibo.it (G.T.); paolo.caraceni@unibo.it (P.C.); 2 Unit of Semeiotics, Liver and Alcohol-Related Diseases, IRCCS Azienda Ospedaliero-Universitaria di Bologna, 40138 Bologna, Italy; maurizio.baldassarre@unibo.it

**Keywords:** cirrhosis, large volume paracentesis, ascites, portal hypertension, TIPS, systemic inflammation, hepatic encephalopathy

## Abstract

The development and progression of ascites represent a crucial event in the natural history of patients with cirrhosis, predisposing them to other complications and carrying a heavy impact on prognosis. The current standard of care for the management of ascites relies on various combinations of diuretics and large-volume paracenteses. Periodic long-term albumin infusions on top of diuretics have been recently shown to greatly facilitate the management of ascites. The insertion of a transjugular intrahepatic portosystemic shunt (TIPS), an artificial connection between the portal and caval systems, is indicated to treat patients with refractory ascites. TIPS acts to decrease portal hypertension, thus targeting an upstream event in the pathophysiological cascade of cirrhosis decompensation. Available evidence shows a significant benefit on ascites control/resolution, with less clear results on patient survival. Patient selection plays a crucial role in obtaining better clinical responses and avoiding TIPS-related adverse events, the most important of which are hepatic encephalopathy, cardiac overload and failure, and liver failure. At the same time, some recent technical evolutions of available stents appear promising but deserve further investigations. Future challenges and perspectives include (i) identifying the features for selecting the ideal candidate to TIPS; (ii) recognizing the better timing for TIPS placement; and (iii) understanding the most appropriate role of TIPS within the framework of all other available treatments for the management of patients with decompensated cirrhosis.

## 1. Introduction

Ascites is the most common decompensating event in patients with cirrhosis, occurring in about 5–10% of compensated patients every year [1]. Moreover, the appearance of ascites also implies a significant worsening in patient prognosis, with a 5-year survival rate dropping from 80 to 50% [2,3]. The transition to refractory ascites (RA), namely ascites that cannot be mobilized or prevented by an appropriate diuretic treatment, results in a markedly increased risk of other complications and hospitalizations, leading to a further worsening in survival, with a 2-year survival rate of 35% [2,3]. The current standard of care for the treatment of clinically relevant ascites relies on the combination of diuretics (both aldosterone antagonists and loop diuretics) and periodic large-volume paracentesis (LVP) when needed, followed by albumin administration according to international guidelines [1,4]. Long-term albumin administration has been proposed on top of the standard of care in this setting [5,6], but its use is still controversial and currently not recommended by international guidelines. When the disease progresses, and refractoriness to treatments develops, the use of Transjugular Intrahepatic Portosystemic Shunt (TIPS) can represent an effective therapeutic approach, due to its ability to reduce portal hypertension, the fundamental pathophysiological mechanism leading to ascites accumulation. Available evidence shows that TIPS can provide a better control of ascites compared to repeated LVPs in selected patients with RA, although the impact on liver transplant-free survival is less clear. In this regard, the criteria currently used to select the candidates for TIPS placement greatly limit the feasibility of this maneuver to a minority of patients with RA. Indeed, multiple factors should be considered—age, severity of liver disease, individual risk of post-TIPS hepatic encephalopathy (HE), and cardiac and renal dysfunction—which can lead to shunt ineffectiveness and an excess of adverse events. Following the introduction of covered stents more than 15 years ago, which has dramatically dropped the incidence of stent failure by antagonizing the occurrence of intra-stent thrombosis, recent research has proposed the use of stents with a reduced diameter, with the aim of improving efficacy and lowering side-effects. However, additional randomized controlled trials (RCTs) are needed to further define patient characteristics, optimal timing, and stent type for TIPS use in the treatment of ascites. The present review discusses the current evidence supporting the use of TIPS for ascites management, along with some uncertainties and emerging perspectives.

## 2. Pathophysiology of Ascites in Patients with Cirrhosis

The current pathophysiological theory explaining the accumulation of ascites in patients with liver cirrhosis integrates the classical hemodynamic hypothesis (based on the concept of the “peripheral arterial vasodilation” [7]) with the more recent evidence on the role of systemic inflammation and immune dysregulation [8] in triggering and sustaining the decompensation of cirrhosis. According to the most recent and comprehensive theory [9], ascites results from the combination of local and systemic pathophysiological factors.

Within the liver, portal hypertension determines an increased pressure gradient between the sinusoids and the interstitial compartment, thus leading to an increased fluid flow towards the lymphatic system tributary to the thoracic duct. Once the draining capacity of the thoracic duct is overwhelmed (it can increase up to ten-fold in patients with severe portal hypertension), intrahepatic lymph accumulates and oozes through the glissonian membrane to the splanchnic cavity. Hypoalbuminemia is generally considered a facilitating factor in this process, due to the reduced plasma oncotic pressure, but evidence is controversial, and experts disagree on its role [10].

Portal hypertension, in combination with local intrahepatic inflammation due to chronic liver damage, also induces the production of vasodilatory substances, such as nitric oxide (NO), endocannabinoids, and carbon monoxide (CO). These substances exert their action at a systemic level, mainly on splanchnic vessels. Splanchnic arterial vasodilatation progressively induces a condition of “effective hypovolemia” and secondary organ hypoperfusion [7]. Compensatory neuro-humoral responses such as the activation of the renin–angiotensin–aldosterone (RAA) axis and sympathetic nervous system (SNS), and arginine–vasopressin (AVP) secretion, stimulate renal fluid retention, thus increasing total blood volume and promoting ascites accumulation. Portal hypertension also alters gut mucosal permeability, favoring the abnormal translocation of bacteria and bacterial products (pathogen-associated molecular patterns, PAMPs) from the intestinal lumen. At the same time, chronic liver damage causes the release of damage-associated molecular patterns (DAMPs) from necrotic hepatocytes. The systemic spread of PAMPs and DAMPs leads to a sustained systemic inflammatory state, triggered by an abnormal activation of the innate immune system [8]. Therefore, recent evidence shows that in the most advanced stages of cirrhosis, inflammation-induced cardiac and renal dysfunction contributes to the further deterioration of effective hypovolemia (by exhausting the compensatory mechanism of increased cardiac output), and to abnormal fluid retention [9].

## 3. Current Standard of Care for Patients with Ascites

Ascites can be assessed and graded from 1 to 3, according to the amount of fluid accumulated in the abdominal cavity. Grade 1 or mild ascites is the amount of fluid only detectable by ultrasound examination. The prognostic implications of this clinical event (included among the “non-acute” decompensating events) are currently not fully understood, nor is it clear if treatments could modify its natural history [11].

In case of grade 2 ascites, current treatment involves a moderate dietary salt restriction and a combined diuretic therapy, with aldosterone antagonists and loop diuretics [1]. Regarding sodium restriction, no clear benefit of low-sodium diets has emerged by clinical trials comparing different dietary regimes. Moreover, an excessive salt reduction can favor a reduced caloric intake and impair nutritional status [12]. It is now clear that patients should only avoid an excessive salt intake [1]. With regard to diuretics, in a pathophysiological perspective, aldosterone antagonists could be used first, when secondary hyperaldosteronism prevails, and loop diuretics could be introduced in long-standing ascites, when tubular sodium reabsorption gains a pre-eminent role [1]. Whether a sequential or a concomitant use of these agents offers the best efficacy has not been clearly established. However, some evidence showed a faster clinical response and lower diuretic-related side-effects (mainly electrolyte imbalance) from adopting a combined approach [13].

When ascites progresses to grade 3 (or severe/tense), periodic LVPs become the treatment of choice. Albumin administration after an LVP (at a dose of 8 g/kg of tapped ascites, especially in the case of drainage of at least 5 L of fluid) is recommended to prevent the development of the so-called post-paracentesis circulatory dysfunction (PPCD) [1].

Refractory ascites (RA) has been defined by the International Club of Ascites (ICA) as “ascites that cannot be mobilized or the early recurrence of which (e.g., after LVP) cannot be satisfactorily prevented by medical therapy” [1,14]. Two different subtypes of RA have been described, according to the lack of response to a maximal diuretic treatment (“diuretic-resistant” ascites) or to the development of diuretic-induced complications that prevent the use of an effective diuretic dosage (“diuretic-intractable” ascites). When refractoriness develops, repeated LVPs followed by albumin administration should be the treatment of choice and diuretic therapy should be discontinued [1]. Since the occurrence of RA carries an abrupt worsening of patient prognosis, the referral of patients for liver transplantation (if feasible) is mandatory.

In recent years, new evidence has shown a potential role for long-term albumin administration in patients with grade 2–3 and even RA [5,6]. Indeed, the ANSWER trial, a multicentric RCT conducted in Italy, showed that the long-term weekly administration of albumin on top of standard diuretic treatment, in patients with uncomplicated grade 2–3 ascites, could significantly ease the management of ascites and improve the overall survival of patients [5]. Another Italian study, performed on a monocentric cohort of patients with RA, showed similar results [6]. Although with non-negligible differences in treatment duration and dose, an important Spanish multicentric RCT (the MACHT trials) contradicted the above-mentioned results [15]. Moreover, long-term albumin use still offers some logistical and economic issues that limit its generalized adoption. Therefore, this promising but debated therapeutic approach is currently not included among the recommendations of international guidelines [16]. However, the last update of the Italian guidelines has included long-term albumin administration among the medical treatment options for ascites [17].

**Figure 1:**
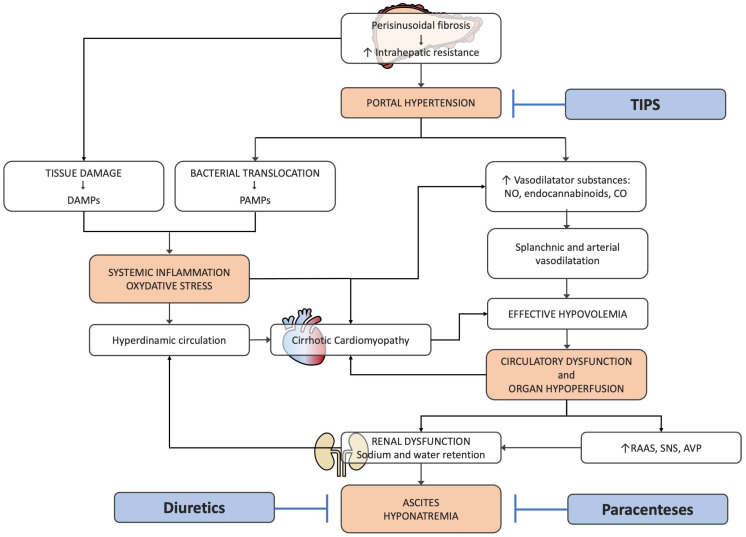
Pathophysiology of decompensation and ascites formation in cirrhosis (see text for details). Diuretics and paracenteses are “symptomatic” treatments, acting on the final events of the pathophysiological cascade. TIPS insertion can counteract the key upstream factor leading to decompensation. TIPS: transjugular intrahepatic portosystemic shunt; DAMPs: damage-associated molecular patterns; PAMPs: pathogen-associated molecular patterns; NO: nitric oxide; CO: carbon monoxide; RAAS: renin–angiotensin–aldosterone system; SNS: sympathetic nervous system; AVP: arginine-vasopressin.

Both diuretics and LVPs, the current standard of care for moderate-to-severe ascites, are only “symptomatic” treatments, which act downstream of the pathophysiological cascade, leading to ascites accumulation, without significantly affecting patient prognosis. Conversely, the insertion of TIPS, namely the creation of an artificial shunt between systemic and portal flow to decrease portal hypertension, represents an approach able to counteract the major upstream pathophysiological mechanism, with a potential impact on the natural history of the disease (Figure 1).

## 4. Technical Aspects and Characteristics of Stent

TIPS insertion opens an artificial shunt between an intrahepatic portal vein branch and a hepatic vein, thus inducing a reduction in the porto-cava pressure gradient (PCPG). In patients with variceal bleeding, the ideal PCPG target to be achieved to effectively prevent re-bleeding has been established as less than 12 mmHg [18]. The optimal PCPG cut-off is not as clear in the case of RA. Indeed, some patients also presented ascites recurrence with a PCPG <12 mmHg, while other patients with PCPG > 12 mmHg after TIPS implantation resolved ascites, suggesting that other factors could be involved in ascites persistence or control [19,20]. At present, no studies were able to identify an optimal PCPG reduction to effectively control ascites. Moreover, most of them were conducted with the currently disused bare metal uncovered stents. The major international guidelines currently recommend the use of self-expanded polytetrafluoroethylene (PTFE)-covered stent-grafts [1,21]. Indeed, PTFE-covered stents showed higher patency, a reduced risk of intra-stent thrombosis, and a better control of ascites, compared to bare metal stents [22,23,24]. Regarding the risk of hepatic encephalopathy (HE) after TIPS placement, available evidence does not clearly show a potentially protective effect of PFTE-covered stents. However, a retrospective study investigating clinical outcomes after PTFE-covered TIPS insertion compared to LVPs plus albumin did not show an increased risk of overt HE [25].

Due to the potentially increased risk of HE, international guidelines recommend a small-diameter stent (namely less than the standard 10 mm diameter) in patients with ascites, but no agreement exists on the optimal diameter [1]. In a study which compared 8 mm and 10 mm stents for the prevention of variceal rebleeding, the former did not increase re-bleedings or stent-dysfunction rates but reduced the incidence of HE and liver function impairment [23]. Another study performed on patients that received 8 mm or 10 mm TIPS for variceal bleeding or RA observed a significantly prolonged survival in patients that underwent smaller stent placement [26]. Therefore, it is currently suggested to prefer the initial insertion of a small-diameter stent (8 mm, but even 6 mm) and progressively dilate it according to the clinical response in controlling ascites and the individual risk of adverse events (HE, cardiac and liver dysfunction). In these cases, reassessment for further dilating the TIPS stent should be performed at intervals of at least 6 weeks [21]. In this regard, the importance of the last generation of the controlled-expansion stent should be underlined. Unlike old grafts, which tend to passively dilate over time, they maintain a fixed diameter. This technical innovation should avoid any uncontrolled increase in the portosystemic shunt and related complications, allowing at the same time a reliable expansion of the stent diameter [27,28]. Whether this new generation of stents could be effectively superior to the previous ones has yet to be defined, although initial results are encouraging.

## 5. Hemodynamic Consequences of TIPS Placement

**Figure 2:**
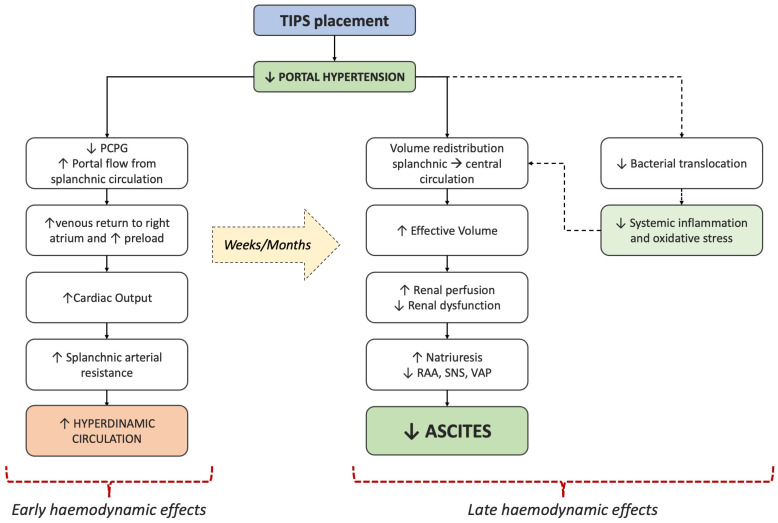
Summary of the main hemodynamic effects of TIPS insertion (see text for details). TIPS: transjugular intrahepatic portosystemic shunt; PCPG: port-cava pressure gradient; CO: carbon monoxide; RAAS: renin–angiotensin–aldosterone system; SNS: sympathetic nervous system; AVP: arginine–vasopressin.

TIPS placement not only exerts local effects on hepatic flow and portocaval pressure but also implies systemic consequences on patient global hemodynamics. The hemodynamic changes occur both during the first hours/days after TIPS placement, and the following months. Early after TIPS insertion, the shunt between the portal and the hepatic vein reduces the PCPG, with a consequent increase in venous return to the right atrium and preload, thus inducing a rise in cardiac output [29,30]. The reduction in portal hypertension also causes an increase in portal flow from splanchnic circulation, which contributes, through TIPS, to a further increase in venous inflow into the right atrium. Thereafter, the resulting increased cardiac output induces a concomitant reduction in peripheral vascular resistance, which leads to a worsening or a new state of the hyperdynamic circulation [29,30] (Figure 2). As a result, the increase in cardiac output does not correspond to an immediate gain in effective blood volume, so arterial blood pressure remains substantially unchanged in the first days after TIPS. Based on the above-mentioned events, it appears essential to exclude pre-existing clinically relevant diastolic dysfunction or pulmonary hypertension before TIPS insertion, as increased venous return could be fatal in compromised cardiac conditions.

Late effects of TIPS are less understood and investigated. During the months following TIPS placement, a reduction in the hyperdynamic state progressively occurs, with a redistribution of the blood volume from the splanchnic bed to the central district and only a minimal increase in cardiac output above the pre-TIPS level [31,32]. The increased effective circulating volume also improves renal perfusion, thus increasing natriuresis, abating the hyperactivation of neurohormonal systems, and improving serum creatinine and estimated Glomerular Filtration Rate (eGFR) [31]. TIPS-induced sodium excretion can be delayed by advanced age and pre-TIPS intrinsic kidney impairment or dysfunction. Some initial evidence suggests that TIPS could also allow a reduction in bacterial translocation from the gut lumen, thus contributing to de-escalating systemic inflammation, another essential pathogenetic component of decompensation in cirrhosis [33] (Figure 2).

## 6. Evidence Supporting the Use of TIPS for Ascites Management

As reported in Table 1, seven RCTs have been published so far on the use of TIPS in the setting of ascites [34,35,36,37,38,39,40]. They all showed a significant improvement in ascites control with TIPS insertion compared to LVPs plus albumin. However, whether TIPS also increases transplant-free survival is less defined and open to discussion. Indeed, only some of the available trials showed an improvement in transplant-free survival [35,38,39]. Among these, we should mention the study published by Narahara et al., which highlighted a superiority of TIPS on survival, but enrolled a very selected population of patients with RA and good liver and kidney function [39]. If we consider the six meta-analyses including the results of available studies, all demonstrated a post-TIPS reduction in ascites, but a concomitant increase in HE episodes [41,42,43,44,45,46]. However, only two of them showed a clear effect of TIPS in increasing transplant-free survival [41,42]. It is also necessary to emphasize some limitations of the available studies. First, almost all of them were conducted before 2010, only using bare-metal stents and not the modern ePTFE-covered stents. Furthermore, most of them enrolled patients with RA, and only a few included some patients with an earlier stage of ascites.

In this regard, we should certainly mention the important study published by Bureau et al. in 2017, which evaluated the use of the ePTFE stent in patients with recurrent ascites [40]. Sixty-two patients with cirrhosis requiring at least 2 (but less than 6) LVPs in the last 3 months were enrolled. During the 12-month follow-up after TIPS insertion, this study reported an improvement in ascites control and a reduction in portal-hypertension-related complications and hospitalizations, without a significant increase in HE episodes. Moreover, a significant improvement in 1-year transplant-free survival for the TIPS group has been shown. However, the severity of ascites of the patients included in the trial deserves some comments. First, the definition of recurrent ascites slightly differs from that proposed by the ICA (namely ascites that recurs at least 3 times within 12 months). Second, the mean number of paracenteses performed in the previous 3 months in enrolled patients was above 4, indicating a severity of ascites that allocate most of patients as having RA.

The major international guidelines currently recommend considering TIPS for both RA and recurrent ascites [1,21]. However, further studies are needed to define the correct timing for TIPS placement (including the threshold of severity for ascites), the impact on long-term survival, and the role of new ePTFE-covered stents with controlled expansion.

**Table 1:** RCTs about TIPS placement for the treatment of ascites.

Author, Year	Setting	Treatment	Main Findings in TIPS Group
Lebrec (1996) [34]	Refractory ascites	Uncovered TIPS vs. LVP	- Higher ascites resolution in Child B pts; - Higher mortality in Child C pts.
Rössle (2000) [35]	Refractory or recurrent ascites	Uncovered TIPS vs. LVP+HA	- Higher 2-year survival; - Better control of ascites; - No differences in HE.
Ginès (2002) [36]	Refractory ascites	Uncovered TIPS vs. LVP+HA	- Lower rate of ascites recurrence; - Lower risk of HRS; - Increased risk of HE; - No differences in mortality.
Sanyal (2003) [37]	Refractory ascites	Uncovered TIPS + SMT (sodium restriction, diuretics, LVP+HA) vs. SMT alone	- Better control of ascites; - Higher risk of HE; - No differences in survival, hospitalization rates, or quality of life.
Salerno (2004) [38]	Refractory or recidivant ascites	Uncovered TIPS vs. LVP+HA	- Higher 1- and 2-year survival; - Better control of ascites; - Higher risk of HE;
Narahara (2011) [39]	Refractory ascites with good renal and liver function	Uncovered TIPS vs. LVP+HA	- Higher 1- and 2-year survival; - Better control of ascites; - Higher risk of HE.
Bureau (2017) [40]	Recurrent ascites (≥2 LVPs in at least 3 weeks)	Covered TIPS vs. LVP+HA	- Higher 1-year survival; - No differences in HE.

TIPS: transjugular intrahepatic portosystemic shunt; LVP: large-volume paracentesis; HA: human albumin; HE: hepatic encephalopathy; HRS: hepatorenal syndrome; SMT: standard medical treatment.

## 7. Patient Selection and Contraindications to TIPS

Although a unanimous agreement is still lacking, only few absolute contraindications to TIPS insertion exist, mainly related to cardiac dysfunction, advanced liver impairment, or structural liver alterations. In particular, the following conditions are generally reported as absolute contraindications to TIPS [21]:

- Severe congestive or valvular heart disease;
- Moderate–severe pulmonary hypertension (assessed with invasive methods) despite an optimized medical treatment;
- Ongoing uncontrolled systemic infection and sepsis;
- Refractory overt HE;
- Unrelieved biliary obstruction;
- Parenchymal liver lesions (e.g., multiple cysts or tumors) that preclude TIPS insertion.

In all other conditions, a comprehensive patient assessment on a case-by-case basis should be performed, and the indication should be discussed in a multidisciplinary setting among hepatologists, interventional radiologists, and surgeons. Indeed, impaired hepatic, cardiac, neurological, and renal function can affect patient prognosis after TIPS or worsen their quality of life and deserve an appropriate discussion. Patient selection for TIPS in the case of ascites is therefore a challenging issue, and multiple factors should be considered, including potentially etiological treatments and the possibility of liver transplantation (LT). The clinical context is completely different from variceal bleeding, which often requires rapid decisions for emergency clinical situations.

Patients with ascites that are potentially eligible for LT should not delay or avoid the assessments aimed at listing [1]. TIPS placement does not affect surgical outcome after LT, but it can determine changes in surgical complexity: on the one hand, a reduction in portal hypertension can ease surgical hemostasis, and on the other, TIPS can also add technical complexity by hindering venous anastomosis. Therefore, it should be considered and discussed with surgeons before insertion, in patients suitable for LT. 

Advanced age can predispose to an increased risk of recurrent or persistent HE and mortality after TIPS. A net age cut-off has not been established, but available studies tend to exclude patients older than 70 years. However, a liver-related mortality prediction model for older people undergoing TIPS has been developed and proposed, although it needs external validation on larger cohorts [47]. A still-open issue, in elderly individuals, is the identification of a specific pre-TIPS evaluation. Whether these patients should routinely undergo a neurological assessment to exclude concomitant neurodegenerative diseases, which could be exacerbated by TIPS insertion, is a debated question that deserves consideration.

One of the major concerns related to TIPS placement is the risk of heart failure. Its incidence ranges from 1% in the first week to 20% in the first year [48], and it can be secondary either to TIPS insertion, due to an increased preload and hyperdynamic circulation, or to pre-existing factors (e.g., valvular diseases, cirrhotic or alcoholic cardiomyopathy, cardiac ischemic disease, pulmonary hypertension). A French study conducted on 100 patients that received TIPS between 2011 and 2016, following a complete cardiac evaluation, tried to identify the predictive factors of post-TIPS heart failure: increased QTc values, aortic stenosis, diastolic dysfunction, left atrial dilatation, and increased pre-TIPS brain natriuretic peptide (BNP) and N-terminal pro-brain natriuretic peptide (NT-proBNP) values [49]. It should be noted that patients with BNP and NT-proBNP in the normal range and an absence of echocardiographic criteria of diastolic dysfunction had minimal risk of post-TIPS heart failure; in contrast, diastolic dysfunction was associated with increased mortality.

TIPS placement should also be carefully evaluated in patients with renal dysfunction. Pre-existing renal impairment can increase the risk of post-TIPS HE and hamper an optimal clinical response in terms of ascites resolution. Renal function and eGFR should be assessed and monitored in all candidates to TIPS, although a clear creatinine threshold contraindicating TIPS has not been identified [21]. However, regarding the role of TIPS as a treatment option in the case of hepatorenal syndrome (HRS), both considering the “old” criteria for type-2 HRS and the more recent definition of HRS–chronic kidney disease (HRS-CKD), available evidence showed a potential benefit both on renal function and survival, but further studies are needed to support these findings [50,51]. With regard to HRS–Acute kidney injury (AKI), a multicenter prospective RCT is currently ongoing to evaluate the efficacy of TIPS within 72 h from HRS-AKI diagnosis, compared to the standard of care with terlipressin and albumin (NCT05346393).

As already reported, the major adverse event of TIPS insertion remains the risk of HE, with the consequent worsening in quality of life. Post-TIPS HE occurs in up to 50% of patients, and almost 10% of cases can require a reduction (or closure) in stent diameter due to the severity or recurrence of clinical symptoms [21]. Once again, the selection of patients plays a key role. The following factors were identified as predictors of HE after TIPS creation: older age, pre-TIPS recurrent or persistent overt HE, impaired liver function, increased creatinine, sarcopenia and hyponatremia [40,52,53]. Covert HE was also predictive of an increased risk of HE after TIPS and, therefore, careful pre-TIPS assessment for subclinical HE is needed. Sarcopenia is a highly prevalent condition in patients with decompensated cirrhosis. Moreover, difficult-to-treat ascites itself (refractory or recurrent) predisposes patients to protein-calorie malnutrition, and a subsequent loss of muscle mass. Sarcopenia is also a known risk factor for HE development after TIPS. At the same time, an improvement in the nutritional status and an increase in muscle mass have been clearly demonstrated following TIPS placement [53,54,55]. Therefore, the challenge to face is the identification of those patients who are too compromised to benefit from TIPS, with a consequent unacceptable increased risk of HE.

Finally, there is no agreement on a defined threshold for liver disease severity, namely the Model for End-stage Liver Disease (MELD) or Child–Pugh score values, for which TIPS could be contraindicated [21]. A recent review tried to summarize all available evidence on TIPS use in patients with cirrhosis and ascites, proposing to identify the ideal patient according to three main factors [56]:

1. Age: <65 years.
2. Liver function: Child–Pugh score ≤ 13; MELD score ≤ 19; no recurrent or persistent HE without precipitants; total bilirubin level < 3 mg/dL; platelet count > 75,000 × 10^9^/L.
3. Cardiac function: no systolic or diastolic dysfunction; no aortic stenosis; normal value of BNP or pro-BNP.

## 8. Conclusions and Future Directions

In case of ascites unresponsive to diuretic therapy, TIPS should be considered an alternative to LVP plus albumin. As mentioned, the first and most important effect of TIPS insertion is the reduction in portal hypertension, a key and upstream event in the pathophysiology of decompensation in cirrhosis. According to the available studies, TIPS placement compared to LVP certainly improves ascites control, with variable results on transplant-free survival, also considering that almost all RCTs have been conducted with old bare stents. In recent years, important technical improvements have been made and implemented in clinical practice: ePTFE-covered stents, smaller-diameter grafts (6 or 8 mm), and controlled-expansion stents. Their consequences in ascites management and long-term survival still deserve to be explored in clinical trials.

Currently, the major experts agree in suggesting the initial insertion of a small (6–8 mm) ePTFE-covered stent and consider over time its gradual dilatation, according to the clinical response on ascites control and the incidence of TIPS-related adverse events. The timeframe and extent of the clinical response (namely the need for paracentesis or moderate-to-high doses of diuretics after TIPS placement) depend on hemodynamic modifications and systemic interactions that vary from patient to patient and should not be considered a failure of TIPS insertion.

Regarding patient selection, some factors assume a primary importance: age, organ functionality (severity of renal, cardiac, or liver dysfunction), risk or history of HE, and sarcopenia. The ideal candidate for TIPS insertion, as well as tools to better stratify patients according to the risk of post-TIPS complications or adverse events, remain to be further explored. The recently proposed Freiburg index of post-TIPS survival (FIPS) combines age, bilirubin, albumin, and creatinine, and it has been shown superior to the major currently available prognostic scores in predicting survival after elective TIPS implantation [57]. It represents a fundamental step toward a better identification of high-risk patients with a worse post-TIPS prognosis. However, it also has some limitations. The proposed prognostic factors do not include clinical features, such as sarcopenia or frailty, which could heavily impact patient clinical course. Further studies should aim to explore further refinements.

From a clinical point of view, other general aspects still deserve further studies: First, the recognition of the stage of ascites that could benefit more from TIPS insertion. Most of the available studies enrolled patients with RA, but it is still not definitely demonstrated if earlier severity stages could obtain better results. Second, the need to include TIPS in the evolving scenario of potential disease-modifying treatments for patients with decompensated cirrhosis, and to better understand its role among the available therapeutic strategies. Third, the use of TIPS in combination with LT. A multidisciplinary discussion of individual cases should aim to optimize the use of TIPS in this setting (to ease surgical procedures during LT, but also to avoid unnecessary TIPS insertion). At the same time, TIPS placement should be encouraged without delay when indicated and feasible, as a potential pathophysiological mechanistic treatment.

## Data Availability

Not applicable.
